# Promoting Freedom and Autonomy in Residential Dementia Care: Lessons from Green Care Farms

**DOI:** 10.1093/geroni/igaf122.3035

**Published:** 2025-12-31

**Authors:** Gijs Steinmann, Maud Hamers, Hilde Verbeek

**Affiliations:** Maastricht University, Maastricht, Limburg, Netherlands; Maastricht University, Maastricht, Limburg, Netherlands; Maastricht University, Maastricht, Limburg, Netherlands

## Abstract

Environmental factors play a crucial role in promoting the functional capacities, well-being, and autonomy of older adults, particularly those with dementia. However, by prioritizing safety and control, traditional nursing homes often create restrictive environments. Green Care Farms (GCFs) are increasingly recognized as a promising alternative, integrating physical, social, and organizational aspects that differ radically from traditional settings. This study examines how residential care environments can be (re)designed to structurally promote the freedom and remaining capacities of people with dementia. Specifically, we identify the environmental aspects through which GCFs foster autonomy and explore their potential implementation in other settings. Our findings are based on qualitative research at two Dutch care settings: a GCF and a regular nursing home (RNH). At the GCF, we combined participant observation with semi-structured interviews to immerse ourselves in residents’ everyday surroundings and identify aspects that enhanced or constrained freedom and autonomy. At the RNH, we conducted participant observation as active members of a project team in which staff sought to integrate GCF elements into their traditional setting. Our analysis reveals that the GCF environment promotes freedom and autonomy through a consistent alignment of physical and organizational aspects. This generates environmental presses that stimulate physical activity to maintain physical functioning, and it enables the acceptance of risks to enhance freedom. In principle, each of these aspects could be implemented in other settings. However, this would require profound organizational changes in RNHs, as staff would need to adopt values and practices that directly oppose traditional ways of working.

